# Rapid Radiofrequency Field Mapping In Vivo Using Single-Shot STEAM MRI

**DOI:** 10.1002/mrm.21676

**Published:** 2008-09

**Authors:** Gunther Helms, Jürgen Finsterbusch, Nikolaus Weiskopf, Peter Dechent

**Affiliations:** 1 MR-Research in Neurology and Psychiatry, Faculty of Medicine, University of GöttingenGöttingen, Germany; 2 Department of Systems Neuroscience, University Medical Center Hamburg-EppendorfHamburg, Germany; 3 Wellcome Trust Centre for Neuroimaging, Institute of Neurology, University CollegeLondon, UK

**Keywords:** radiofrequency mapping, stimulated echo, human brain

## Abstract

Higher field strengths entail less homogeneous RF fields. This may influence quantitative MRI and MRS. A method for rapidly mapping the RF field in the human head with minimal distortion was developed on the basis of a single-shot stimulated echo acquisition mode (STEAM) sequence. The flip angle of the second RF pulse in the STEAM preparation was set to 60° and 100° instead of 90°, inducing a flip angle-dependent signal change. A quadratic approximation of this trigonometric signal dependence together with a calibration accounting for slice excitation-related bias allowed for directly determining the RF field from the two measurements only. RF maps down to the level of the medulla could be obtained in less than 1 min and registered to anatomical volumes by means of the *T*_2_-weighted STEAM images. Flip angles between 75% and 125% of the nominal value were measured in line with other methods. Magn Reson Med 60:739–743, 2008. © 2008 Wiley-Liss, Inc.

MRI at static field strengths higher than 1.5T suffers from inhomogeneity of the RF field as the in vivo wavelength approaches the typical dimensions of the human body. Deviation of the local flip angle from its nominal value may affect local contrast and signal amplitude. Quantitative parameter estimation, in particular of *T*_1_, must therefore consider a suitable correction (1).

The straightforward way to determine the local flip angle is to vary the nominal flip angle and determine the local value from a fit of the signal equation. In most cases this requires prior knowledge of the relaxation times in tissue, a prerequisite that may not be met in vivo, in particular in the presence of pathologies. Four different strategies have been reported to reduce the influence of local tissue properties: 1) imaging under (almost) fully relaxed conditions to eliminate the influence of *T*_1_-dependent saturation (2); 2) the double-echo method with a specific flip angle dependence but negligible relaxation dependence (3–5); 3) use of compensation pulses (6) or purging pulses (7); and 4) to determine the point of signal elimination, e.g., achieved by a 180° excitation pulse in a spoiled gradient echo sequence (8). Safety limits for the specific absorption rate render the latter method unfeasible at higher field strengths. The time required for full relaxation precludes the use of nonselective pulses in multislice MRI, and thus the mapping of the RF field without slice profile effects (9).

In this article we propose a rapid, low-power method applicable at high and ultrahigh field strengths. It capitalizes on the dependence of the stimulated echo acquisition mode (STEAM) signal on the second (“flip-back”) pulse. The effect of the slice-profile on the flip angle dependence was calibrated by comparing the slice-selective and nonselective implementation in a single slice. Implementation as a dual-angle technique with multislice single-shot STEAM MRI provided anatomically reliable 3D maps of the flip-angle distribution across the whole intracranial space in less than a minute.

## THEORY

Deviations of the local flip angle α(x) from its nominal value, α_nom_, are primarily due to insufficient flip angle adjustment, transmitter coil inhomogeneity, or effects of the dielectric and conductive sample. The local mismatch can be described by a spatially dependent factor, the transmit bias factor, *f*_T_ = *f*_T_(x):


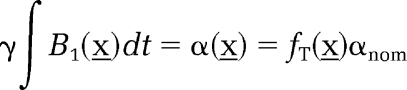
[1]

(with flip angles given in radian units). This alters the flip angle dependence of the MR signal, which is commonly stated for the flip angles conforming to theoretical assumptions. In reality, the measured signal, *S*, depends on the local flip angle, but only the nominal value is known. For the basic situation of on-resonance nonselective excitation after full relaxation, the effect of *f*_T_ is observed as a spatially dependent shift of the signal maximum in the sinusoidal dependence on α_nom_ (5):


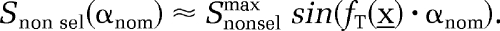
[2]

An additional source of error may occur with slice-selective excitation when the slice profile changes with the flip angle. The theoretical sinusoidal signal dependence is then altered by the integrated slice profile (10). Due to increasing contributions from the flanks, the signal maximum is shifted to a flip angle slightly larger than π/2 and major positive deviations from the sinus occur when the flip angle approaches π. However, below a certain threshold flip angle the signal from the slice can be described in good approximation by a sinus, as shown using STEAM localization (11). In the context of this work, the shift of the maximum from π/2 to α_max_ has to be accounted for. This is achieved by introducing a slice profile-dependent factor (*f*_slice_ = π/2α_max_):


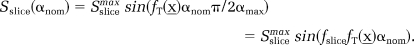
[3]

Note that *f*_slice_ is smaller than 1 as the maximum is shifted toward higher flip angles. Its value can be determined by fitting Eq. [3] once the transmit bias factor (that is, the local flip angle) is known.

The factor in the argument of the sinus should be determined from points around the signal maximum. Low flip angles result in a correlation of the arbitrary signal amplitude (*S*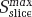) and *f*_T_ as well as low signal-to-noise. In this work the signal was approximated around its maximum (attained at *f*_T_ α_nom_ = α_max_) by a quadratic polynomial:


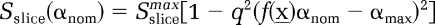
[4]

The parameters α_max_ and *q* depend on the pulse shape, but not on the local RF field. The values of *q* may slightly differ from √1/2 (for α_max_ in radians). Again, they can be determined when α(x) corresponds to the nominal value or when *f*_T_ is known (see Calibration of Slice Parameters, below). With the knowledge of α_max_ and *q* it is then possible to calculate arbitrary local values of *f*_T_ from the signals of two STEAM images (*S*_1_ and *S*_2_) at different nominal flip angles (α_1_ and α_2_) by:


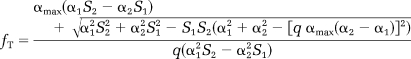
[5]

The solution of the quadratic problem of Eq. [4] is detailed in the Appendix. Measurement at two flip angles is sufficient because the use of inverse trigonometric functions with nonunique solutions (5) is avoided.

## MATERIALS AND METHODS

### Experimental

The study was performed on a 3T whole-body clinical MR system (Magnetom Trio, Siemens Medical Solutions, Erlangen, Germany) equipped with a 40 mT/m gradient system and a 35 kW RF power amplifier for the T/R bodycoil. Using the bodycoil for transmission and an eight-channel phased-array receive coil for reception the method was tested on a healthy adult volunteer who gave informed written consent in accordance with the Helsinki convention as supervised by the local review board. A quality assurance phantom for MR spectroscopy (MRS) (18 cm sphere, General Electric Medical Systems, Milwaukee, WI) was examined using the transmit-receive headcoil (16 rod birdcage resonator, 30 cm diameter).

**FIG. 1:**
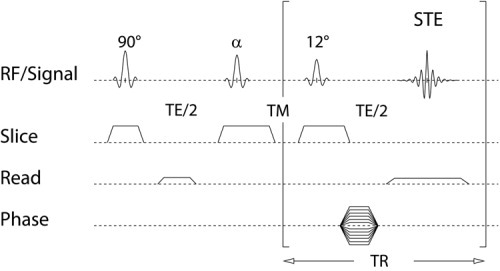
Sequence diagram of the single-shot STEAM sequence The flip angle of the second RF pulse (α) was varied, while those of excitation and readout were kept constant.

A 64 × 52 matrix with a field-of-view of 224 × 182 (3.5 mm resolution) was acquired using a full-Fourier single-shot STEAM sequence with centric reordering of *k*-space lines (12) (Fig. 1). The standard 90° pulse shape (se2560_a90) of 1.28 ms duration was used for slice-selective excitation and flip-back. A Hamming-filtered sinc-pulse with time-bandwidth product of 2 was used for rapid readout with a bandwidth of 200 Hz per pixel at a TR of 8 ms (TE = 6 ms). A volume of 52 interleaved slices (2.8 mm thickness and 0.7 mm gap) was acquired within 22.4 sec.

For the two-point measurement of *f*_T_ at 3T, flip angles of α_1_ = 60° and α_2_ = 100° were chosen on the basis of our experiments that *f*_T_ may vary between 0.75 and 1.25 across the human brain at this specific field strength (see Results). For comparison, 3D RF-maps were measured by means of a fast low angle shot (FLASH)-based dual-TR method (14) (α/TE/TR1/2 = 45°/2.2/14/54 ms) at the same resolution (3.5 mm isotropic) in 2:08 min.

### Calibration of Slice Parameters

In the context of this article, “calibration” will refer to the determination of the nonlinear function relating the slice-selective signal to the local flip angle. In principle, it may be performed with a homogeneous transmit coil on a nondielectric and nonconductive liquid (ε_r_ ≈ 1) and thus *f*_T_(x) ≈ 1. However, measurements on the manufacturer-provided Luxor oil phantom were unstable, probably due to viscosity-related motion artifacts. As in Ref. (5), the calibration was performed on a manufacturer-supplied spherical phantom of 18 cm diameter containing an aqueous solution of 1.25 mg NiSO_4_ × 6 H_2_0 per liter. This phantom had been designed to exhibit a conductive load and RF inhomogeneities similar to those observed in vivo in a human head. The flip angle was increased to 120° in steps of 10° under fully relaxed conditions (TR = 22.4 sec). To determine the transmit bias (Eq. [2]), the flip angle dependence was measured in a single slice with a nonselective flip-back pulse (zero slice-selection gradient). In addition, the sinc-shaped pulse was used for flip-back in order to demonstrate the influence of the pulse shape (10,14,15). The signals were averaged in a homogeneous region-of-interest (ROI) around the signal maximum at the center of the sphere. The parameters of the quadratic approximation were then determined from seven data points around the signal maximum taking into account the transmit bias. Nonlinear least-square curve fitting of ROI intensities was performed with numerical estimation of the derivatives using KaleidaGraph 3.6 (Synergy Software, Reading, PA).

### Image Postprocessing

The sets of dicom images were converted to 3D volumes in analyze format for further processing using the FSL 3.2 software package (provided by the Centre for Functional Magnetic Resonance Imaging of the Brain, University of Oxford, UK, http://www.fmrib.ox.ac.uk/fsl). After linear registration the images were low-pass-filtered by a Gaussian kernel (full-width-at-half-maximum = 10 mm). Maps of *f*_T_ were then calculated from Eq. [5] using the parameters α_max_ and *q* determined in the calibration. The dual TR 3D-FLASH volumes were processed as described previously (13).

## RESULTS

**FIG. 2:**
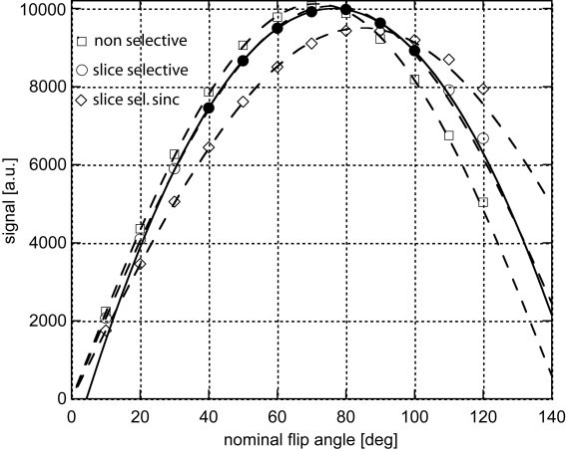
Calibration of signal dependence. Fitting a sinusoidal dependence (dashed line) to the nonselective experiment (square symbols) yielded the transmit bias of 1.262 ± 0.003. Thus, the slice factor of the optimized RF pulse (circles) and the sinc RF pulse (diamond symbols) could be determined (dashed lines). Due to systematic deviations at 110° and 120°, these data points have been omitted from the fit. The bold line demonstrates the quality of the quadratic approximation (fitted to seven data points around the signal maximum depicted as solid circle symbols). Good correspondence with the sinusoidal dependence was found between 40° and 125° (corrected flip angle values).

The calibration experiments are shown in Fig. 2. The peak in the sinusoidal signal curves (dashed) appeared at lower nominal flip angles than 90°, since the actual flip angle was increased by the large transmit bias (*f*_T_ = 1.262 ± 0.003). In contrast to the nonselective case (square symbols), shifts of the maximum and positive residues were observed for the slice-selective signals (circle symbols: optimized RF pulse; diamond symbols: filtered sinc pulse). Hence, the two largest flip angles were omitted from the fit. The slice factor and signal amplitude of the sinc pulse (*f*_slice_ = 0.841 ± 0.003) were smaller than those of the optimized excitation pulse (*f*_slice_ = 0.939 ± 0.003) The quadratic approximation (solid line and symbols) described the signal dependence excellently in the flip angle range between 30° and 105°, that is, between 40° and 125° in corrected values. The fit of the quadratic function to the seven points around the maximum yielded *q* = 0.6315 ± 0.0014 and α_max_ = 1.6797 ± 0.0012, which was consistent with *f*_slice_ within fitting errors.

**FIG. 3:**
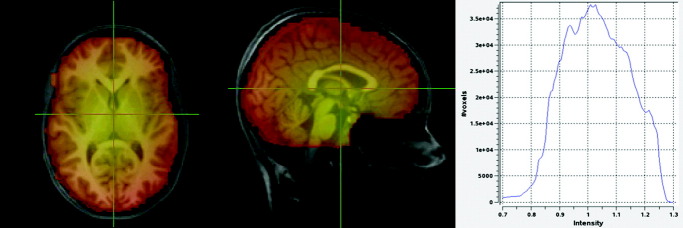
RF-map in vivo axial (**a**) and sagittal (**b**) cross-sections of the in vivo RF map and the corresponding distribution of *f*T (**c**). The opaque color-overlay required linear registration to a 3D structural dataset. Note the high flip angles in the central brain regions and the low flip angles at the cortex.

Figure 3a,b shows a color overlay of the 3D *f*_T_ map after linear registration to an anatomical *T*1-weighted volume. The highest values (yellow) are found in the central region of the basal ganglia, thalamus, and the pons. The typical range of *f*_T_ across the brain (indicated by the histogram in Fig. 3c) was between 0.75 and 1.25. The choice of nominal flip angle ensured that the actual flip angles fell into the interval where the quadratic approximation is valid.

**FIG. 4:**
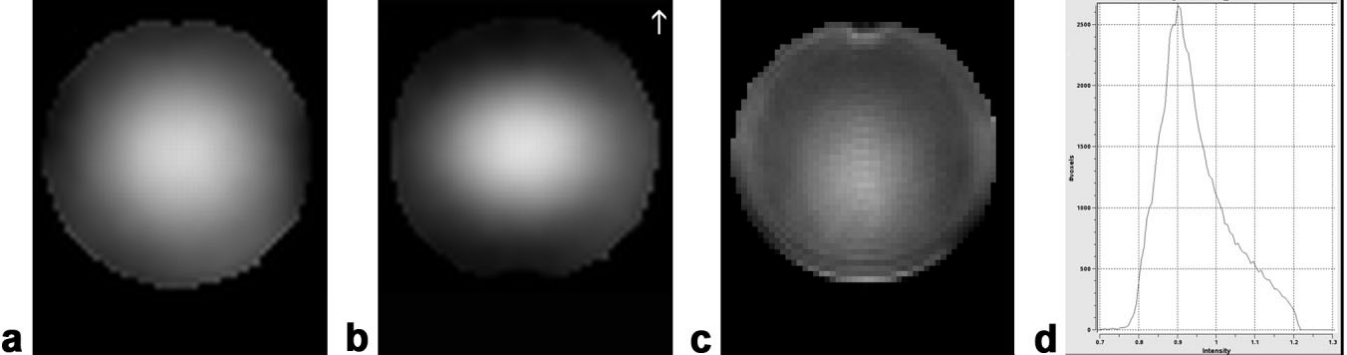
RF map in a spherical phantom axial (**a**) and sagittal (**b**) cross-sections of the in vivo RF map in the MRS phantom (*f*T windowed between 0.75 and 1.25) and the corresponding distribution of *f*T (**d**) co-registered and up-sampled to 1 mm resolution. Comparison with the dual-TR FLASH method at original 3.5 mm resolution (**c**) shows systematic RF-dependent variations and residual ringing pattern (ratio windowed between 0.8 and 1.2).

Figure 4 shows the maps obtained on the MRS phantom. The circular-polarized headcoil induced some lateral flip angle inhomogeneities on the axial cross-section the MRS phantom (Fig. 4a) that were not seen with body coil excitation. Yet this did not visibly affect the spatial distribution of *f*_T_ (Fig. 4d). As predicted (16), the RF inhomogeneities were larger perpendicular to the direction of the applied RF, that is, along the z-direction. This is seen on the sagittal cross-section (Fig. 4b). For comparison with the dual-TR FLASH method the ratio of the corresponding maps at the original resolution of 3.5 mm is shown in Fig. 4c on the cross-section corresponding to Fig. 4a. On average, the dual-TR maps yielded 5% lower estimates. At the center of the phantom the dual-TR FLASH method yielded a flip angle of 54.2°; the single-shot STEAM method 56.2°; that is, a difference of 3.6%.

## DISCUSSION

The proposed method for mapping the RF field combines three novel features. First, the use of a single-shot STEAM sequence to reduce spatial distortions compared to echo-planar imaging (EPI); second, the transformation of the slice-selective signal to nonselective excitation by means of a calibration experiment; third, the use of a quadratic approximation. The latter can be solved for a two-angle measurement because the use of inverse trigonometric functions and their nonuniqueness can be avoided. By means of the flip angle dependence, the *transmitted* RF component (*B*_1+_) was mapped and expressed as a transmit bias factor to correct the nominal flip angle specified in the pulse sequence. A comprehensive review of RF mapping techniques is given in Ref. (17).

RF mapping using single-shot STEAM can be regarded as an alternative to the 3D double-echo method based on EPI (5), since it only suffers minimally from geometric distortions, even at high fields. For example, undistorted maps were obtained even in the orbitofrontal cortex and down to the level of the medulla and cerebellum (Fig. 3b). At 3T the signal losses from the 12° stimulated echo and from repeated nonselective refocusing are of similar degree. We observed slightly higher values of *f*_T_ than reported in Ref. (5) both in the calibration and in vivo. In contrast to methods using nonselective RF pulses (5,13), residual *T*_1_-relaxation effects were avoided in our study by interleaved multislice acquisition at TR/2 = 12 ms. On the other hand, calibration, approximation, and the two-angle implementation may bias the results systematically. This bias is exacerbated if a wider range of flip angles is encountered, e.g., in larger objects or ultrahigh field strength. In this case, Eq. [3] has to be solved directly and cannot be approximated. The quadratic approximation may also be used for nonlinear fitting to multiple flip angles to reduce the influence of motion artifacts in singular images. The time required for a two-angle measurement was 48 sec, that is, half the time required by the dual-echo EPI (≈2 min) and dual-TR FLASH methods.

Of the two 90° RF pulses in the single-shot STEAM sequence, we arbitrarily chose to vary flip angle of the second. For symmetric pulse shapes the flip-back profile (single quantum coherence to longitudinal pathway) is identical to the excitation profile (15). The varying excitation profile of the flip-back pulse is superimposed on the one of the 90° excitation pulse. Thus, the estimates of the slice factor cannot be transferred immediately to a single slice-selective pulse.

Nevertheless, the calibration approach may be applied to any pulse in sequence run under fully relaxed conditions. Omitting the slice-profile effect (*f*_slice_) would result in an underestimation of *f*_T_ by about 7%. The transmit bias may be used to correct parameter estimates obtained with nonselective pulses, in particular *T*_1_ (1). The slightly *T*_2_-weighted STEAM images have to be used to register the RF field maps to other anatomical images that require transmit bias correction, to overcome the lack of anatomical structure in the RF field. By accounting for the shift of the slice-selective signal, single-shot STEAM yields anatomically reliable RF-maps of the human brain down to the medulla in less than a minute.

## APPENDIX

Compared to the trigonometric equation (Eq. [3]), the quadratic approximation (Eq. [4]) can be solved for *f*_T_ if the signals (*S*_1_ and S_2_) for just two nominal flip angles (α_1_ and α_2_) are known. The transmit bias factor, *f*_T_, is introduced into Eq. [4] via α(x). The arbitrary amplitude, *S*, is cancelled by division of *S*_1_ = *S*(α_1_) and *S*_2_ = *S*(α_2_):

[A1]

This expansion is rewritten as a quadratic equation in *f*_T_:

[A2]

Solving the nonstandard form *C* = *f*_T_ 2*q*^2^*B* −  *q*^2^*A* for *f*_T_ by quadratic expansion yields then one positive solution:

[A3]

The root expression is simplified by sorting first for terms containing *q*^2^ α_max_ and then for *S*_1_*S*_2_ to derive Eq. [5].
